# Invasome: A Novel Nanocarrier for Transdermal Drug Delivery

**DOI:** 10.3390/nano10020341

**Published:** 2020-02-17

**Authors:** Soraya Babaie, Azizeh Rahmani Del Bakhshayesh, Ji Won Ha, Hamed Hamishehkar, Ki Hyun Kim

**Affiliations:** 1 Biotechnology Research Center, Student Research Committee, and Faculty of Advanced Medical Science, Tabriz University of Medical Sciences, Tabriz 51368, Iran; 2 Stem Cell and Regenerative Medicine Institute, and Department of Tissue Engineering, Faculty of Advanced Medical Sciences, Tabriz University of Medical Sciences, Tabriz 51368, Iran; 3 School of Pharmacy, Sungkyunkwan University, Suwon 16419, Korea; ellenha2@gmail.com; 4 Drug Applied Research Center, Tabriz University of Medical Sciences, Tabriz 51368, Iran

**Keywords:** transdermal, liposome, invasome, terpene, nanocarrier

## Abstract

Invasomes are novel vesicular systems that exhibit improved transdermal penetration compared to conventional liposomes. These vesicles contain phospholipids, ethanol, and terpene in their structures; these components confer suitable transdermal penetration properties to the soft vesicles. The main advantages of these nanovesicles lie in their ability to increase the permeability of the drug into the skin and decrease absorption into the systemic circulation, thus, limiting the activity of various drugs within the skin layer. In this paper, several features of invasomes, including their structure, mechanism of penetration, applications, characterization, and potential advantages in dermal drug delivery, are highlighted. Overall, this review suggests that enhanced transdermal penetration of drugs using invasomes provides an appropriate opportunity for the development of lipid vesicular carriers.

## 1. Introduction

The transdermal route is an important pathway for localized or systemic effects [1]. The stratum corneum, the outer layer of the skin, is an essential skin permeation barrier for many drugs. To overcome this barrier, several techniques have been developed [2,3,4,5,6], including the use of methods that change the stratum corneum (SC) continuity, such as ultrasound, electroporation, and iontophoresis, and the use of the vehicle and nanocarriers to improve drug penetration [3,5,7,8,9]. Recently, different types of nanocarriers have been designed to improve the dermal and transdermal delivery of medicines. Vesicular systems appear to be suitable carriers owing to their physicochemical properties, such as deformability, size, and charge, which can be modified by altering lipid constituents and preparation methods [10,11]. Liposomal vesicular systems can incorporate both lipophilic and hydrophilic drugs to assist in the penetration of the incorporated agents [12]. However, conventional liposomes are not approved as appropriate systems for transdermal delivery of drugs as they are unable to permeate the inner layers of skin and, therefore, their effects remain limited to the upper layers [13]. Novel elastic vesicles containing penetration enhancers are superior to conventional liposomes due to their improved interactions with skin and better drug penetration [14,15]. The primary deformable or elastic vesicles, named Transfersomes^®^, were developed by Cevc et al. in the 1990s. These vesicles are composed of phospholipids and edge activators, such as polysorbate or sodium cholate, producing elastic carriers for improved transdermal drug delivery [16,17,18,19,20]. The encouraging results seen with Transfersomes^®^ led to the development of other novel elastic vesicles via alterations in the vesicular composition. In previous examinations, elastic vesicles such as niosome (prepared mostly by non-ionic surfactant and cholesterol) [21] and ethosome (containing high amount of ethanol in their structure) [22] have displayed potential as a drug carrier. Invasomes are novel and flexible vesicles containing a mixture of soy phosphatidylcholine (PC), terpenes, lyso PC, and ethanol with improved skin penetration in comparison with liposomes. Furthermore, invasomes have the same structural constituents as liposomes but contain terpene in their structure. Terpenes are hydrocarbon compounds and are known to be the primary constituents of essential oils from many plants. Addition of terpenes creates deformable vesicles, which can increase the fluidity of the lipid bilayers of the skin [23,24,25,26]. The ability to permeate through skin layers enhances the activity of invasomes, which exert their effects by fluidizing the bilayer structure of SC lipids and disturbing lipids and intracellular protein interactions [27]. Although there are comprehensive review literatures about topical dermal and transdermal drug delivery [28] and literatures about invasomes [29], this review deals with various aspects of invasomes with new organization and includes the latest studies about invasome.

## 2. Invasomes in Comparison with Liposomes

**Figure 1:**
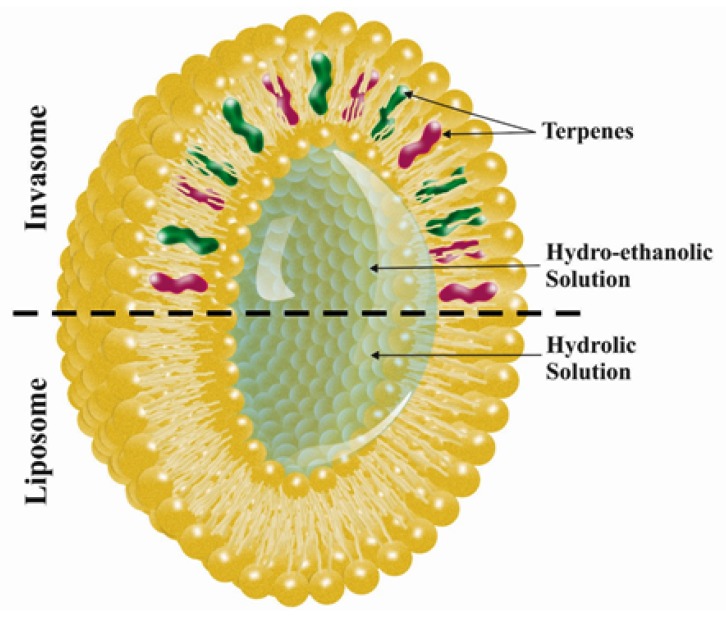
Invasome versus liposome.

**Figure 2:**
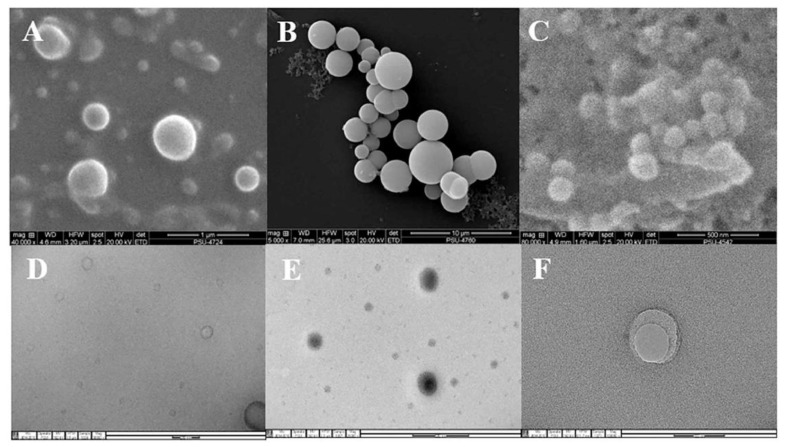
SEM photographs of liposome (**A**), invasome (**B**), and transfersome formulations (**C**), and TEM photographs of liposome (**D**), invasome (**E**), and transfersome formulations (**F**). Reprinted with permission from reference [33]. Copyright 2018, Elsevier.

Liposomes are phospholipid-based vesicular structures composed of anionic, cationic, and neutral lipids and cholesterol that improve the encapsulation of lipophilic, hydrophilic, and amphiphilic drugs. Lipophilic drugs are placed in the inner part of the lipid bilayer, hydrophilic drugs in the aqueous core, and amphiphilic types in the interlayer of the vesicles [30,31]. Contrary to this, invasomes are flexible liposomes consisting of phospholipids, ethanol, and one terpene molecule or a mixture of terpenes. Ethanol increases the fluidity of lipids in the vesicle structure, creating a soft structure less rigid than conventional liposomes and, therefore, enhancing its permeability into the skin [32]. Similarly, terpenes have also been shown to improve penetration by disrupting the tight structure of the SC lipids [24] (Figure 1). Microscopic image of invasome and its differences from liposome and transfersome are shown in Figure 2. SEM photographs indicated that all vesicles displayed smooth surface and spherical structure (Figure 2A–C). TEM photographs showed the surface morphology of liposomes and invasomes were unilamellar (Figure 2D,E), while unilamellar to multilamellar was revealed in the case of transfersomes (Figure 2F).

## 3. Penetration Mechanism of Invasomes

**Figure 3:**
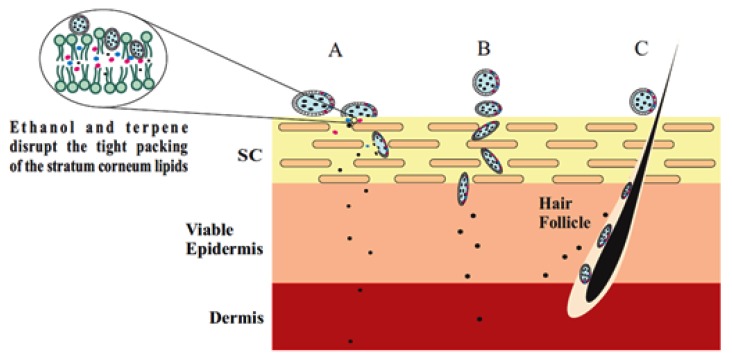
Penetration mechanism of invasomes through the stratum corneum (SC). Enhanced penetration (**A**), intact penetration (**B**), and trans-appendageal penetration (**C**).

Terpenes and ethanol in the invasomes cause deformability of the vesicles, disrupt the SC bilayer skeleton, and act as penetration enhancers, improving the permeability of the invasomes [34]. According to Dragicevic-Curic et al. during penetration of the invasome, one part of the vesicle disintegrates and releases its components, such as terpenes, phospholipid segments, and single phospholipid molecules, which enhance the penetration and fluidize the SC lipids. Smaller invasome vesicles, which do not disintegrate, penetrate through the SC intact [35]. According to Verma and group, upon penetration, intact invasomes may reach the inner parts of the SC by the follicular transport pathway or via the narrow hydrophilic channels existing in the intercellular region of the SC [17]. Honeywell-Nguyen et al. revealed that smaller intact invasomes can penetrate the deeper part of the SC through the channel-like areas. This was deduced by the flexible vesicles of various sizes that were discovered at the channel-like areas in the deeper layer of the SC and skin surface vesicles [36,37]. In general, a number of invasomes disintegrate when penetrating the SC, whereas smaller vesicles and flexible invasomes penetrate the deeper layers intact [38] (Figure 3).

## 4. Effect of Composition on the Physicochemical Characteristics of Invasomes

### 4.1. Effect of Ethanol

The addition of ethanol in the formulation of lipid nanovesicles is an effective strategy to increase the fluidity of the lipid bilayer of the skin [39,40]. The interaction of ethanol with the lipid elements in the polar group area of the SC leads to alterations in the structure of the keratinized or lipophilic domains, decreased transition temperature of lipids, and consequently fluidization and disruption of the tightly packed SC lipids [40,41]. Ethanol-based nanocarriers can fluidize and disturb the SC lipids [42]. The presence of ethanol increases the flexibility of the intercellular lipid matrix due to the rotating freedom of the lipid acyl chains. Thus, ethanol increases the fluidity of lipids in the vesicle structure, resulting in a structure that has softer and less rigid properties than conventional liposomes [32]. In addition to enhanced penetration ability, ethanol creates a net negative surface charge and limited vesicle aggregation due to electrostatic repulsion, leading to increased stability of invasomes under storage conditions [38,43,44,45].

### 4.2. Effect of Terpenes

#### 4.2.1. Effect of Terpene on Penetration

X-ray diffraction and differential scanning calorimetry (DSC) results showed that terpenes lead to increased drug penetration by disrupting the tight bilayers and lipid packing in the SC [46]. Furthermore, breaking the hydrogen bonds and extracting SC lipids [47], enhancing the partition into the SC by improving lipid fluidity [48] and increasing diffusion via the intercellular lipids [49] are another mechanisms that have been reported to increase drug permeability by terpenes.

Dragicevic-Curic et al. revealed that various types of terpenes have a synergistic effect on the permeation of temoporfin. Invasomes containing a 1% mixture of three types of terpenes (citral, cineol, and limonene) demonstrated higher temoporfin permeability than invasomes containing 1% citral alone [35]. In another study, Dragicevic-Curic et al. demonstrated the relationship between the permeated amount of temoporfin and the amounts of terpenes in the invasomes. They indicated that vesicles containing 1% terpenes have a 1.7-fold higher temoporfin penetration effect than vesicles containing 0.5% terpenes. Therefore, incorporation of temoporfin in vesicles containing 1% terpenes could lead to deeper penetration [38].

#### 4.2.2. Effect of Terpene on the Size of the Invasomes

Examination of particle size demonstrated that the size of the invasomes is directly correlated to the amount of terpenes; the size of the invasomes increases as the amount of terpene increases. The size of vesicles containing 1% terpenes was 124 nm, whereas the size of vesicles with 0.5% terpenes was 93.0 nm [38]. Prasanthi et al. showed that the size of finasteride-loaded invasomes was influenced by the molecular size of terpene and the concentration of the added terpene. The size of invasomes containing nerolidol (molecular size 222 g/mol) was around 11 to 13 µm. The vesicle sizes of nimesulide-loaded liposomes containing citral, limonene, and cineole were 194 nm, 216 nm, and 244 nm, respectively [50,51].

#### 4.2.3. Effect of Terpene on the Shape of the Invasomes

The results of cryo-transmission electron microscopy (cryo-TEM) were in agreement with the DSC and ESR results, indicating the influence of terpenes on the shape of the invasomes, i.e., in addition to spherical vesicles, malformed vesicles of varied shapes also existed in invasomal dispersions [38]. Dragicevic-Curic et al. used cryo-electron microscopy to observe the lamellarity and shape of invasomes with various percentages of terpenes. Their results revealed that invasomes with 0.5% terpenes were mostly unilamellar and bilamellar or oval and spherical in shape; however, in the invasomal formulation with 1% terpenes, the invasomes appeared to be unilamellar and bilamellar. Therefore, the combination of 1% terpenes with the invasomes increased the membrane elasticity of invasomes, the percentage of terpenes, and the amount of deformed vesicles [38].

### 4.3. Synergistic Effects

A synergistic effect between phospholipids, ethanol, and terpenes on dermal absorption has been visibly observed [38]. Dragicevic-Curic et al. suggest that one part of the invasome disintegrates during permeation in the upper layers of skin and releases the phospholipids and terpenes, which act as permeation enhancers that fluidize the intercellular lipids. Furthermore, the ethanol in the invasome fluidizes the intercellular lipids and enhances the penetration of flexible vesicles [52,53]. Verma et al. indicated that invasomes increased the transdermal permeation of cyclosporine A compared to an ethanolic solution. The improved efficiency of invasomes compared to an ethanolic solution suggests a synergistic effect of phospholipid, terpenes, and ethanol. Dragicevic-Curic et al. demonstrated that the improved permeation of temoporfin (mTHPC) with 1% terpenes was due to the concentration of terpenes and the synergistic effects of terpenes and ethanol [38]. Thus, the results from the aforementioned studies [38,54,55] point toward the synergistic effect of phospholipid, terpenes, and ethanol in the reformed activity of invasomes in comparison with liposomes. 

## 5. Invasome Stability

The storage temperature has a significant effect on the physical stability of invasomes, i.e., the size of the particles and the polydispersity index (PDI) value. During storage at room temperature, all invasomes show an increase in the particles size and the PDI value, demonstrating physical instability, i.e., aggregation or fusion of the vesicles [38,52]. In the case of the Dragicevic-Curic et al. study, the PDI of the invasomes stored at 4 °C was stable during storage for 12 months; however, after six months of storage, the invasomes showed a significantly increased particles size and PDI value [38]. With regard to drug content, Lakshmi et al. determined that there was a loss of 10% of the encapsulated drug after one month of refrigeration. The loss of encapsulated drug increased to 50% when stored at room temperature [56].

## 6. Potency Versus Toxicity of Terpenes as One of the Main Components of Invasome

Different approaches have been examined to progress the transdermal permeation of drugs. Among them, use of effective and safe classes of penetration enhancers such as natural terpenes are the most popular methodology. Despite proper performance of penetration enhancers in transdermal delivery, few of them have been approved for clinical application due to their skin irritation and toxicity. In general, there is balance between the potency of penetration enhancers and their toxicity. Terpenes present high penetration potency for both hydrophilic and lipophilic compounds even at small concentrations. The strategic mechanism for terpene penetration occurs through interaction with SC intercellular lipids [57]. Terpenes are applied alone or in combination with other drug delivery systems as permeation enhancers for therapeutic applications [58,59,60,61]. But beside the potency of terpenes in comparison to different penetration enhancers, the toxicities of terpenes should be considered. The toxicity assessment of terpenes in two skin cell lines using 3-(4,5-dimethylthiazol-2-yl)-2,5-diphenyltetrazolium bromide (MTT) assay and evaluation of transepidermal water loss (TEWL) indicated that terpenes achieved from natural sources are usually considered to be safe in comparison to synthetics [62].

## 7. Applications of Invasome

In this section, we have enumerated several applications of invasomes. An overview of various studies on the therapeutic applications and skin permeability enhancement of invasomes is given in Table 1 and Table 2, respectively.

Hypertension is a global series health problem because of its association with cardiovascular disease. Advances in the treatment of hypertension have played a major role in decrease of stroke mortality and coronary heart diseases [71]. Isradipine is administered for the management of hypertensive diseases. This drug has vast first-pass metabolism and low oral bioavailability. Thus, Qadri et al. attempted the development of an invasomal formulation of isradipine using β-citronellene (terpene). It was concluded that the isradipine-loaded invasomes improved the transdermal penetration of the drug for the potential management of hypertension [72]. In another study, Kamran et al. studied the antihypertensive effect of olmesartan loaded invasomes. The examinations revealed that the bioavailability of olmesartan improved 1.15 times in the nano-invasomes formulation in comparison to the control formulation in Wistar rats [47].

Hyperpigmentation disorders arise because of melanin overproduction, leading to unusual distribution of melanin in specific portions of the skin. Control of tyrosinase activity is the rate limiting step for melanin production. Phenylethyl resorcinol is a novel skin whitening agent, which prevents the tyrosinase activity. Lower stability and poor water solubility are the major limitations of phenylethyl resorcinol for cosmetic products. Amnuaikit et al. prepared topical formulation of phenylethyl resorcinol loaded invasomes and transfersomes and compared them with conventional liposome. In vitro assessment demonstrated better melanin content reduction and tyrosinase inhibition activity for invasomes and transfersomes in comparison with liposome [33].

Acne is a prevalent chronic inflammatory infection of the skin that has various negative effects on young adults. It creates emotional stress, discomfort, and long-lasting scarring to the skin [73]. Dapsone is a special sulfone with efficacy against acne. It is completely absorbed after administration in the oral form. Thus, the topical application of dapsone is predicted to be active in the treatment of mild to moderate acne conditions. El-Nabarawi et al. prepared dapsone-loaded invasomes with different terpenes (cineole, limonene, citral, and fenchone) to examine their ability to deliver dapsone through the skin. Their investigation revealed that the deposition of dapsone in the skin is significantly improved by invasomes [59]. 

Photodynamic therapy is one of the interesting approaches, including the use of photosensitizing compound, which concentrates in the target cells and then locally irritates by visible light. Finally target cell will be impaired by apoptosis or necrosis [74]. Dragicevic-Curic et al. prepared an invasomal formulation of temoporfin, a lipophilic photosensitizer with insufficient skin penetration, and compared its skin penetration depth with that of a conventional liposomal formulation. Invasomes were prepared using ethanol (3.3% w/v), lecithin (10% w/v), and a blend of terpenes (citral, cineole, and D limonene in 0.5 and 1% w/v). Their findings demonstrated that invasomes comprising a 1% blend of terpenes exhibited higher temoporfin deposition in the SC compared to the liposomes. The temoporfin loaded in the invasomes with 1% terpenes was found to reach the inner parts of the skin [38].

Benign prostatic hyperplasia is a common disease which occurs in elderly mans. The development of the prostate leads to pathological change in the urine and kidney [75]. In the Prasanthi and Lakshmi et al.’s study, finasteride was chosen as a model drug to assess the in vivo transdermal delivery of invasomes using the iontophoretic technique. The invasomes formulation consisted of terpenes (nerolidol, carvone, and limonene) using Taguchi’s robust design method for optimization. Among several formulations, a sample containing limonene (0.5%) showed 21-fold better penetration than the controls. Furthermore, the optimized formulation was tested for improved permeation using the iontophoretic technique. Here, the permeation was 26-fold higher than that of the control. Finally, the gel form of the optimized formulation was tested and compared to an oral suspension. The results demonstrated that invasomes are effective carriers for transdermal delivery of finasteride through the application of iontophoretic techniques [50].

Studies have shown that the invasome is one of the successful drug delivery systems for hydrophilic drugs. According to an in vitro study by Badran et al., invasomes containing a 1% mixture of terpenes were more efficient in the dermal delivery of hydrophilic compounds than an aqueous solution. These vesicles were also efficient in combination with a Dermaroller^®^ (a new microneedle device), which further improved drug permeation and penetration [50].

## 8. Conclusions

Invasomes are non-toxic vesicular carriers that have been used for transdermal delivery. They can also assist in the penetration of the incorporated agent, while incorporating both lipophilic and hydrophilic drugs. Phospholipids, ethanol, and terpenes are the main units of the invasome structure. Furthermore, the penetration of these vesicles can be simply modulated by modifications of their composition. Based on the available studies, we emphasize the high potential of invasomal formulations in topical therapeutic applications. These systems expand the opportunities for dermatology research in the future. Nevertheless, additional studies are necessary to demonstrate the clinical efficacy of invasomes. With regard to the mentioned ability of invasomes as drug carriers, we also suggest a focus on the development of appropriate technology for the industrial production of invasomes as will be necessary for the application of invasomes in the treatment and prevention of diseases in future dermatology research.

## Figures and Tables

**Table 1:** Therapeutic application of invasomes.

Drug	Applications	Type of Study	Study Outcomes	Ref.
Avanafil	Treatment of erectile dysfunction	Excised abdominal rat skin	Optimized invasomal film improved the bioavailability and transdermal permeation of Avanafil	[63]
Idebenone Azelaic acid	Antioxidant/anticancer, anti-acne	Excised human skin	LeciPlex exhibited higher permeation of idebenone and invasomes exhibited higher permeation of azelaic acid	[64]
Curcumin	Anti-inflammatory, antioxidant, and anticancer activity	Shed snake skin	Physicochemical characteristics of the formulations influenced by terpene and Tween 20	[65]
Curcumin	Anti-inflammatory, anti-carcinogenic, Etc.	Excised rat skin	Invasome with 0.5% limonene improved intradermal penetration of curcumin	[56]
Temoporfin	Photodynamic therapy (a pilot study)	Mice skin	Temoporfin invasomes containing a 1% terpene mixture decreased tumor size significantly by photodynamic therapy compared to control groups	[35]
Temoporfin	Photodynamic therapy	Human epidermoid tumor cell line A431	In the A431 cells temoporfin-loaded invasomes were more cytotoxic	[66]
Temoporfin	Photodynamic therapy	Abdominal human skin	Invasomal formulation with 1% mixture of terpenes exhibited a significantly enhanced deposition of temoporfin in the SC compared to liposomes	[38]
Ferulic acid	Antioxidant effect	Excised human skin	Ethosomes are better vesicular carriers for the delivery of ferulic acid into the skin than invasomes	[67]

**Table 2:** Enhanced skin permeability of invasomes.

Drug	Applications	Type of Study	Study Outcomes	Ref.
Nitroxide TEMPO	Measuring the antioxidative capacity	Excised human skin/excised porcine skin	Invasomes improved measurement times of antioxidative capacity by two-fold	[68]
Fluorescent label	Tracking of invasomes	Excised human skin human forearm skin	Strong spectroscopic evidence shows deep penetration of intact invasomes in the SC	[2]
Temoporfin	Photosensitizer	ESR measurements	Terpenes improved the fluidity of the bilayers, whereas temoporfin reduced the fluidity. Therefore, invasomes represent vesicles with excessive membrane flexibility	[55]
3-Carboxy-2,2,5,5-tetramethyl-1-pyrrolidinyloxy (PCA)	Spin-labeling compound	Excised porcine skin	PCA permeation was improved 2.5-fold for CMS and two-fold for invasomes in comparison with PCA solution	[69]
Carboxyfluorescein Temoporfin	Hydrophilic model drug, lipophilic model drug	Excised human skin	Ethosomes and invasomes increased the delivery of hydrophilic drug, for example carboxyfluorescein, into the deep layers of skin	[23]
Calceine Carboxyfluorescein	Low-molecular weight hydrophilic model drugs	Excised human skin	Calcein penetration improved two- and seven-folds by transfersomes and invasomes, respectively	[70]
